# The development of an extended Weibull model with applications to medicine, industry and actuarial sciences

**DOI:** 10.1038/s41598-024-61308-8

**Published:** 2024-05-29

**Authors:** Muhammad Imran, Najwan Alsadat, M. H. Tahir, Farrukh Jamal, Mohammed Elgarhy, Hijaz Ahmad, Arne Johannssen

**Affiliations:** 1 Department of Statistics, The Islamia University of Bahawalpur, Bahawalpur, 63100 Pakistan; 2 Department of Quantitative Analysis, College of Business Administration, King Saud University, P.O. Box 71115, 11587 Riyadh, Saudi Arabia; 3 Department of Basic Sciences, Higher Institute for Administrative Sciences, Belbeis, Al-Sharqia Egypt; 4 Mathematics and Computer Science Department, Faculty of Science, Beni-Suef University, Beni-Suef, 62521 Egypt; 5 Near East University, Operational Research Center in Healthcare, TRNC Mersin 10, Nicosia, 99138 Turkey; 6 Center for Applied Mathematics and Bioinformatics, Gulf University for Science and Technology, Mishref, Kuwait; 7 Department of Computer Science and Mathematics, Lebanese American University, Beirut, Lebanon; 8 Faculty of Business Administration, University of Hamburg, 20146 Hamburg, Germany

**Keywords:** COVID-19, Cancer, Complementary Bell-G family, Group acceptance sampling plan, Quality control, Risk measures, Biological techniques, Cancer, Mathematics and computing

## Abstract

This paper delves into the theoretical and practical exploration of the complementary Bell Weibull (CBellW) model, which serves as an analogous counterpart to the complementary Poisson Weibull model. The study encompasses a comprehensive examination of various statistical properties of the CBellW model. Real data applications are carried out in three different fields, namely the medical, industrial and actuarial fields, to show the practical versatility of the CBellW model. For the medical data segment, the study utilizes four data sets, including information on daily confirmed COVID-19 cases and cancer data. Additionally, a Group Acceptance Sampling Plan (GASP) is designed by using the median as quality parameter. Furthermore, some actuarial risk measures for the CBellW model are obtained along with a numerical illustration of the Value at Risk and the Expected Shortfall. The research is substantiated by a comprehensive numerical analysis, model comparisons, and graphical illustrations that complement the theoretical foundation.

## Introduction

The Weibull model is the most commonly used model for survival and reliability analysis in many domains, however, it is less suitable when the data show a non-monotonic failure rate. This is because traditional models have a number of drawbacks. Various distributions have been frequently used for modeling actuarial and econometric data, but they often failed to offer a sufficient fit. However, model flexibility can be improved through generalization, and this practice has become quite common in recent times. Various new generalizations, new families of distributions and parameter induction approaches not only enrich the statistical literature but also enable researchers and practitioners to choose different flexible models to achieve better fits. The readers are referred to Lee et al.^1^, Maurya and Nadarajah^2^, Tahir and Cordeiro^3^ and Tahir and Nadarajah^4^ for a detailed discussion and various parameter induction approaches in baseline models. The compounding approach is frequently used as it presents new models by combining two or more similar or dissimilar models, taking into account the nature of the random variable(s) and their supports.

There are two different approaches that can be used in the compounding method: one is based on the zero-truncated power series distribution, and the other uses zero-truncated continuous lifespan models. Using the zero-truncated Poisson, geometric, logarithmic, binomial, negative-binomial or the power series distribution has two main benefits: (1) there are seldom any instances of the value zero in real data sets, so one have to treat the value zero as being excluded, and (2) the number of complementary risks for component failures is the basis on which the compounding process is developed and this number must therefore be larger than or equal to one. Here we discuss the Poisson-G class, because its structure closely resembles and can be compared to our proposed class of distributions. Since the Poisson distribution is a widely used discrete model for count data, its compound models are also equally studied in the continuous situation. It is all because of its versatility and simplicity in practical use^2^. The cumulative distribution function (cdf) of the complementary Poisson-G (CP-G) class for series structure by considering a truncated random variable is given by

1 $$\begin{aligned} F\left( x\right) =\frac{e^{\lambda G\left( x\right) }-1}{e^{\lambda }-1}, \end{aligned}$$

where $\lambda$ is the parameter of the Poisson distribution and *G*(.) is the cdf of any baseline or parent model. Note that Castellares et al.^5^ achieved somewhat better fits than the Poisson model using a discrete Bell distribution (DBellD) constructed from well-known Bell numbers^6^, see the probability mass function (pmf)

2 $$\begin{aligned} \text {P}(X=x)=\frac{\lambda ^{x}e^{-e^{\lambda }+1}B_{x}}{x!},\qquad \qquad x=0,1,2,\ldots , \end{aligned}$$

where $B_{x}$ are the Bell numbers. The DBellD possesses a number of beneficial characteristics, such as a single parameter distribution. The Poisson model cannot be nested into the Bell model despite both belonging to the one-parameter exponential family of distributions. However, for small values of the parameter, the Bell model tends to the Poisson distribution, and the DBellD is also infinitely divisible. These DBellD properties inspired the development of its generalized class, compared mathematically and empirically to the CP-G class and its particular models. Fayomi et al.^7^ expanded the DBellD and produced its generalized class, the exponentiated Bell-G (EBell-G) family. The cdf for the EBell-G family of distributions is provided by^7^ as

3 $$\begin{aligned} F\left( x\right) =\frac{1-e^{-e^{\lambda }\left( 1-e^{-\lambda G^{\theta }(x)}\right) }}{1-e^{1-e^{\lambda }}}, \end{aligned}$$

where $\lambda$ and $\theta$ represent the Bell and shape parameter, respectively. In the case of complementary and competing risks, Algarni^8^ proposed its complementary version. In many cases, information regarding a specific factor that caused the failure is unavailable, and the only information provided is the lifetime of the maximum or minimum among all the risk factors. Such phenomena regularly occur in various fields such as reliability, biology, data science, actuarial sciences, and health care.

In this paper, we study and revisit the extended Weibull model in connection with the CBell-G family of distributions ^8^. In particular, we develop the complementary Bell Weibull (CBellW) model, derive its properties and discuss various practical applications. The proposed CBellW model has some of the characteristics listed below:

- It is more flexible then the well-known complementary Poisson Weibull model.
- It is tractable, has three parameters and comparatively simple probability density function (pdf) and cdf.
- It has a very good fit for heavy-tailed and skewed data.
- It works well when the Weibull, exponential or Burr distribution is used as baseline model, and the failure rate function can have various shapes, including unimodal, upside-down bathtub, increasing or decreasing shapes.

Note that the Weibull distribution is also an important lifetime distribution and there are several recent modifications^9–11^.

The paper is organized as follows. “The CBellW distribution and its properties” section presents the CBellW model and its key distributional properties. “Simulation study” section discusses the results of the simulation study, while “Real-life applications” section focuses on various applications of the CBellW model using six real data sets. Finally, the paper concludes in “Concluding remarks” section.

## The CBellW distribution and its properties

### General distributional properties

Practitioners can employ the CBellW distribution to analyze various types of data because of the failure rate function’s versatility. Consider the baseline cdf and pdf for the Weibull distribution, $G\left( x\right) =1-\exp \left( -\left( \frac{x}{\alpha }\right) ^{\beta }\right)$ and $g\left( x\right) =\frac{\beta }{\alpha ^{\beta }}\left( x\right) ^{\beta -1}\exp \left( -\left( \frac{x}{\alpha }\right) ^{\beta }\right) ,$ for $x>0$, $\alpha >0$ and $\beta >0$, respectively. Then, the cdf of the CBellW distribution is as follows:

4 $$\begin{aligned} F(x;\lambda ,\,\alpha ,\,\beta )=\frac{\exp \left( e^{\lambda \left[ 1-\exp \left( -\left( x/\alpha \right) ^{\beta }\right) \right] }-1\right) -1}{\exp \left( e^{\lambda }-1\right) -1}, \end{aligned}$$

where $x>0$, $\alpha >0$ and $\beta >0$. The pdf corresponding to Eq. (4) is as follows:

5 $$\begin{aligned} f(x;\lambda ,\,\alpha ,\,\beta )= & {} \lambda \,\frac{\beta }{\alpha ^{\beta }}\left( x\right) ^{\beta -1}\exp \left( -\left( x/\alpha \right) ^{\beta }\right) \,\exp \left[ \lambda \,\biggl (1-\exp \left[ -\left( x/\alpha \right) ^{\beta }\right] \biggr )\right] \\{} & {} \exp \left( e^{\lambda \Bigl (1-\exp \left[ -\left( x/\alpha \right) ^{\beta }\right] \Bigr )}-1\right) \,\left[ \exp \left( e^{\lambda }-1\right) -1\right] ^{-1}\nonumber . \end{aligned}$$

The survival function related to the CBellW distribution is as follows:

6 $$\begin{aligned} S(x;\lambda ,\,\alpha ,\,\beta )=\frac{\exp \left( e^{\lambda }-1\right) -\exp \left( e^{\lambda \,\Bigl (1-\exp \left[ -\left( x/\alpha \right) ^{\beta }\right] \Bigr )}-1\right) }{\exp \left( e^{\lambda }-1\right) -1}. \end{aligned}$$

**Figure 1:**
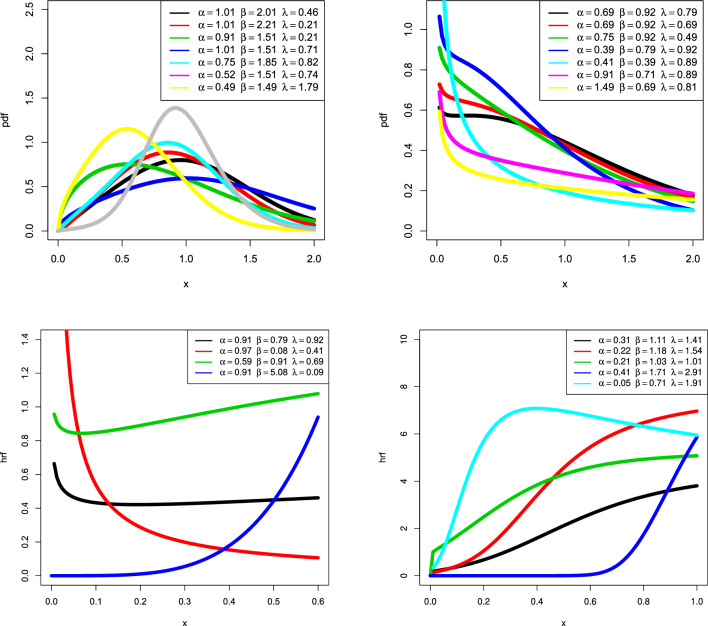
Plots of pdf and hrf of the CBellW for different parameter values.

**Figure 2:**
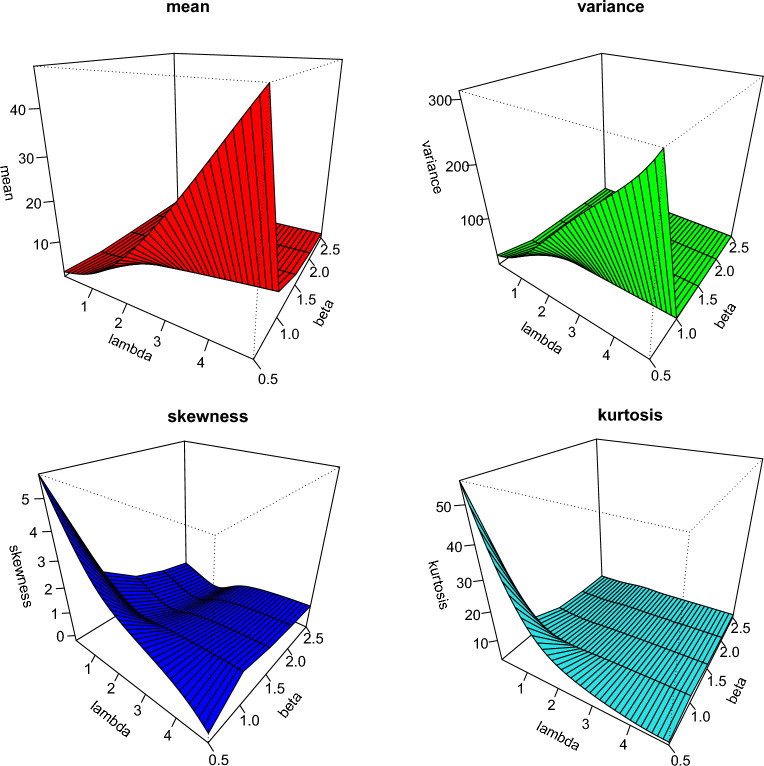
Graphical illustration of mean, variance, skewness and kurtosis of the CBellW model for different parameter values.

The hazard rate function (hrf) is the ratio of $\frac{f(x)}{1-F(x)}$ and can be obtained using Eqs. (5) and (6). The quantile function (qf) of the CBellW distribution is as follows:

7 $$\begin{aligned} Q_{G}\left( z\right) =\alpha \Biggl (-\log \left[ 1-\Biggl \{\lambda ^{-1}\,\log \left[ 1+\log \left\{ 1+u[\exp (e^{\lambda }-1)-1]\right\} \right] \Biggr \}\right] \Biggr )^{1/\beta }, \end{aligned}$$

where *u*[0,1]. As the qf has a closed form solution, it can be used to obtain *L*-moments, and it is suitable to design a GASP as well as various actuarial risk measures. Figure 1 demonstrates the possibility of symmetric, reversed-J, and right-skewed for the pdf of the CBellW distribution. In general, after a failure of different engineering systems the hrf initially has to drop, then it is reasonably static, and lastly, there is a growing failure rate. The terms “burning,” “random,” and “wear-out failure zones” refer to these three phases in reliability theory. The hrf plots have some adaptable shapes, such as increasing, decreasing, and increasing–decreasing shapes, which quantify the characteristics of the lifetime distribution. It can represent the second phase of the bathtub-shaped failure rate because it has a long constant failure rate period as shown in Fig. 1, whereas Fig. 2 shows the mean, variance, skewness and kurtosis of the CBellW model. By increasing $\lambda$, the mean and variance tend to increase. On the other hand, skewness and kurtosis reduce when $\lambda$ increases. The scale parameter $\alpha$ is considered as 1.

#### Proposition 1

The pdf of the CBellW distribution can be expressed in the form

8 $$\begin{aligned} f(x)=\sum _{n=0}^{\infty }t_{n}\pi \left[ x;\left( n+1\right) \alpha ,\beta \right] , \end{aligned}$$

where $$t_{n}=\sum _{v=0}^{\infty }\zeta _{v}\left( v+1\right) \left( -1\right) ^{n}\left( {\begin{array}{c}v\\ n\end{array}}\right)$$ and $\zeta _{v}$ is defined is Eq. (43) (see Appendix) and the last term $\pi \left[ x;\left( n+1\right) \alpha ,\beta \right] =\frac{\beta }{\alpha ^{\beta }}\left( x\right) ^{\beta -1}\exp \left( -\left[ n+1\right] \left( x/\alpha \right) ^{\beta }\right)$ is the Weibull pdf.

#### Proof

Using Eq. (41) yields

9 $$\begin{aligned} f(x)=\sum _{v=0}^{\infty }\zeta _{v}\left( v+1\right) \,\frac{\beta }{\alpha ^{\beta }}\left( x\right) ^{\beta -1}\exp \left( -\left( x/\alpha \right) ^{\beta }\right) \left( 1-\exp \left[ -\left( x/\alpha \right) ^{\beta }\right] \right) ^{v}, \end{aligned}$$

and by applying binomial expansion to the last term we get

$$\begin{aligned} {\left( 1-\exp \left[ -\left( x/\alpha \right) ^{\beta }\right] \right) ^{v}=\sum _{n=0}^{v}\left( -1\right) ^{n}\left( {\begin{array}{c}v\\ n\end{array}}\right) \exp \left[ -n\left( x/\alpha \right) ^{\beta }\right] ,} \end{aligned}$$

and the above expression reduces to

$$\begin{aligned} {f(x)=\sum _{n=0}^{v}\sum _{v=0}^{\infty }\zeta _{v}\left( v+1\right) \left( -1\right) ^{n}\left( {\begin{array}{c}v\\ n\end{array}}\right) \,\frac{\beta }{\alpha ^{\beta }}\left( x\right) ^{\beta -1}\exp \left[ -\left[ n+1\right] \left( x/\alpha \right) ^{\beta }\right] .} \end{aligned}$$

This gives the desired results and completes the proof of Proposition 1. $\square$

The general result of Proposition 1 shows that the CBellW pdf is a linear combination of Weibull densities. Therefore, several mathematical properties of CBellW can be derived from those of the Weibull distribution. Some of them will be presented below.

### Ordinary and incomplete moments

The mean and variance of the CBellW distribution can be obtained by using Eq. (10), where $\text {mean}=\mu _{1}^{\prime }$ and $\text {variance}=\mu _2=\mu _{2}^{\prime }-\left( \mu _{1}^{\prime }\right) ^{2}.$ Moreover, the first four moments can be obtained using the well-established relationship between ordinary and central moments. The moment-based measure of skewness and kurtosis, respectively, is obtained by using $\beta _{1}=\frac{\mu _{3}^{2}}{\mu _{2}^{3}}$ and $\beta _{2}=\frac{\mu _{4}}{\mu _{2}^{2}}$, where $\mu _{3}=\mu _{3}^{\prime }-3\mu _{2}^{\prime }\mu _{1}^{\prime }+2(\mu _{1}^{\prime })^{3}$ and $\mu _{4}=\mu _{4}^{\prime }-4\mu _{3}^{\prime }\mu _{1}^{\prime }+6\mu _{2}^{\prime }(\mu _{1}^{\prime })^{2}-3(\mu _{1}^{\prime })^{4}$. Pearson’s coefficient of skewness and kurtosis can be yielded as $\sqrt{\beta _{1}}$ and $\beta _{2}-3$, respectively. The *r*th raw or ordinary moment of the CBellW distribution is given by

10 $$\begin{aligned} \mu _{r}^{\prime }=\mathbb {E}(X^{r})=\alpha ^{r}\Gamma \left( \frac{r}{\beta }+1\right) \sum _{n=0}^{\infty }t_{n}\frac{1}{\left( n+1\right) ^{1+\frac{r}{\beta }}}, \end{aligned}$$

where $t_n$ is as in Proposition 1. On the other hand, there are many important and useful applications for incomplete times. For instance, they are essential when calculating the average waiting time, deviation, conditional moments, measures of income disparity, etc. The representation of the *r*th incomplete moments is provided by $\mu _{s}\left( x\right) =\int _{-\infty }^{t}x^{s}f(x)dx$. Using Eq. (41), we get

11 $$\begin{aligned} \mu _{s}\left( x\right) =\mathbb {E}(X^{s}1_{\{X\le x\}})=\alpha ^{s}\sum _{n=0}^{\infty }\frac{t_{n}}{(n+1)^{s/\beta +1}}\Gamma \left( \frac{s}{\beta }+1,\left[ n+1\right] \left( x/\alpha \right) ^{\beta }\right) , \end{aligned}$$

where $t_n$ is given in Proposition 1, and $\Gamma \left( a,b\right)$ is the Gamma function.

### Moment generating function

In probability theory and statistics, several statistical measures are used to specify the distribution of interest namely the moment generating function (mgf), characteristic function, the *r*th moments, qf, etc. Let *X* be a random variable associated to *f*(*x*) given in Eq. (8). The mgf is defined by $E\left( e^{tx}\right) =\int e^{tx}f\left( x\right) dx$. Here, we use the Wright generalized hypergeometric function,

12 $$\begin{aligned} _{p}\Psi _{q}\left[ \begin{array}{cc} \left( \alpha _{1},A_{1}\right) ,\ldots , &{} \left( \alpha _{p},A_{p}\right) \\ \left( \beta 1,B_{1}\right) ,\ldots , &{} \left( \beta _{p},B_{p}\right) \end{array};x\right] =\sum _{n=0}^{\infty }\frac{\Pi _{j=1}^{p}\Gamma \left( \alpha _{j}+A_{j}n\right) }{\Pi _{j=1}^{q}\Gamma \left( \beta _{j}+B_{j}n\right) }\frac{x^{n}}{n!}, \end{aligned}$$

to derive the mgf. Considering

13 $$\begin{aligned} f(x;\lambda ,\alpha ,\beta )=\sum _{n=0}^{\infty }t_{n}\frac{\beta }{\alpha ^{\beta }}\left( x\right) ^{\beta -1}\exp \left[ -\left( \Omega \,x\right) ^{\beta }\right] dx, \end{aligned}$$

for computational ease we set $\Omega =\frac{\left( n+1\right) ^{1/\beta }}{\alpha }$, and by expanding the series $e^{tx}=\sum _{m=0}^{\infty }\frac{t_{m}}{m!}x^{m}$, we obtain

$$\begin{aligned} M\left( t\right) =\frac{\alpha ^{\beta }}{\Omega ^{\beta }}\sum _{n=0}^{\infty }t_{n}\sum _{m=0}^{\infty }\frac{(\frac{t}{\Omega })^{m}}{m!}\Gamma \left( m/\beta +1\right) . \end{aligned}$$

Hence, we have the following expression for the mgf:

14 $$\begin{aligned} M\left( t\right) =\frac{\alpha ^{\beta }}{\Omega ^{\beta }}\sum _{n=0}^{\infty }t_{n}\,\,_{1}\Psi _{0}\left[ \begin{array}{cc} 1,1/\beta \\ - \end{array};\frac{t}{\Omega }\right] . \end{aligned}$$

### Reliability

Numerous applications related to reliability have been conducted in various fields. We are able to calculate the failure probability at a specific time point due to aspects of reliability. Let $X_1$ and $X_2$ be two random variables that follow the CBellW distribution. If the applied stress is more than the component’s strength, it will fail; but, if $X_1>X_2$, it will operate satisfactorily. Here, we derive the reliability of the CBellW model when $X_{1}$ and $X_{2}$ are independent with $f(x;\lambda _{1},\alpha ,\,\beta )$ and $F(x;\lambda _{2},\alpha ,\,\beta )$ as well as identical scale $(\alpha )$ and shape $(\beta )$ parameters. It is then given by

$$\begin{aligned} R=\intop _{0}^{\infty }f_{1}\left( x\right) \,F_{2}\left( x\right) \,dx. \end{aligned}$$

By using Eqs. (41) and (42), we get

$$\begin{aligned} f(x;\lambda _{1},\alpha ,\,\beta )=\sum _{v=0}^{\infty }\zeta _{v}\left( \lambda _{1}\right) \,\left( v+1\right) \,\left\{ \frac{\beta }{\alpha ^{\beta }}\left( x\right) ^{\beta -1}\exp \left[ -\left( x/\alpha \right) ^{\beta }\right] \right\} \,\left\{ 1-\exp \left[ -\left( x/\alpha \right) ^{\beta }\right] \right\} ^{v} \end{aligned}$$

and

$$\begin{aligned} F(x;\lambda _{2},\alpha ,\,\beta )=\sum _{t=0}^{\infty }\zeta _{t}\left( \lambda _{2}\right) \left\{ 1-\exp \left[ -\left( x/\alpha \right) ^{\beta }\right] \right\} ^{t+1}, \end{aligned}$$

where $\zeta _v$ is defined in Eq. (43), so it holds

$$\begin{aligned} R=\sum _{v=0}^{\infty }\zeta _{v}\left( \lambda _{1}\right) \,\left( v+1\right) \,\sum _{t=0}^{\infty }\zeta _{t}\left( \lambda _{2}\right) \,I\left( \alpha ,\,\beta ,v,\,t\right) \end{aligned}$$

with

$$\begin{aligned} I\left( \alpha ,\,\beta ,v,\,t\right) =\intop _{0}^{\infty }\left\{ \frac{\beta }{\alpha ^{\beta }}\left( x\right) ^{\beta -1}\exp \left[ -\left( x/\alpha \right) ^{\beta }\right] \right\} \,\left\{ 1-\exp \left[ -\left( x/\alpha \right) ^{\beta }\right] \right\} ^{v+t+1}dx. \end{aligned}$$

Applying the binomial expansion and by simplifying, we get

$$\begin{aligned} {I\left( \alpha ,\,\beta ,v,\,t\right) =\sum _{z=0}^{v+t+1}\left( -1\right) ^{z}\left( {\begin{array}{c}v+t+1\\ z\end{array}}\right) \intop _{0}^{\infty }\frac{\beta }{\alpha ^{\beta }}\left( x\right) ^{\beta -1}\exp \left[ -\left[ z+1\right] \left( x/\alpha \right) ^{\beta }\right] dx,} \end{aligned}$$

$$\begin{aligned} {I\left( \alpha ,\,\beta ,v,\,t\right) =\sum _{z=0}^{v+t+1}s_{z}\left[ z+1\right] ^{-1},} \end{aligned}$$

where $$s_{z}=\left( -1\right) ^{z}\left( {\begin{array}{c}v+t+1\\ z\end{array}}\right) .$$

### Residual and reversed residual life

The *n*th moment of the residual life of *X* is given by

$$\begin{aligned} m_{n}\left( t\right) =\frac{1}{1-F\left( t\right) }\intop _{t}^{\infty }\left( x-t\right) ^{n}dF\left( x\right) . \end{aligned}$$

By using Eq. (8), one gets

$$\begin{aligned} m_{n}\left( t\right) =\frac{1}{1-F\left( t\right) }\sum _{p=0}^{\infty }t_{p}^{*}\frac{\beta }{\alpha ^{\beta }}\intop _{t}^{\infty }x^{r}\left( x\right) ^{\beta -1}\exp \left[ -\left[ n+1\right] \left( x/\alpha \right) ^{\beta }\right] dx, \end{aligned}$$

where $t_{p}=t_{n}$ and $$t_{p}^{*}=t_{p}\sum _{r=0}^{n}\left( {\begin{array}{c}n\\ r\end{array}}\right) \left( -t\right) ^{n-r}$$. Here, the mean residual life of *X* can be achieved by setting $n=1$ in Eq. (15).

15 $$\begin{aligned} m_{n}\left( t\right) =\frac{1}{1-F\left( t\right) }\sum _{p=0}^{\infty }t_{p}^{*}\frac{\alpha ^{r}}{\left[ n+1\right] ^{\frac{r}{\beta }+1}}\gamma \left( \frac{r}{\beta }+1,\left[ n+1\right] \left( t/\alpha \right) ^{\beta }\right) , \end{aligned}$$

where the function $\gamma (a,b)$ represents the upper incomplete Gamma function. The following expression gives the *n*th moment of reversed residual life:

$$\begin{aligned} M_{n}\left( t\right) =\frac{1}{F\left( t\right) }\intop _{0}^{t}\left( t-x\right) ^{n}dF\left( x\right) , \end{aligned}$$

16 $$\begin{aligned} {M_{n}\left( t\right) =\frac{\beta }{\alpha ^{\beta }F\left( t\right) }\sum _{p=0}^{\infty }t_{p}^{**}\intop _{0}^{t}x^{r}\left( x\right) ^{\beta -1}\exp \left[ -\left[ n+1\right] \left( x/\alpha \right) ^{\beta }\right] dx,} \end{aligned}$$

where $$t_{p}^{**}=t_{p}\sum _{r=0}^{n}\left( {\begin{array}{c}n\\ r\end{array}}\right) \left( -1\right) ^{r}t^{n-r}$$. Then, the mean reverse residual life or mean inactivity time of *X* can be obtained by setting $$n=1$$ in Eq. (17):

17 $$\begin{aligned} M_{n}\left( t\right) =\frac{1}{F\left( t\right) }\sum _{p=0}^{\infty }t_{p}^{**}\frac{\alpha ^{r}}{\left[ n+1\right] ^{\frac{r}{\beta }+1}}\gamma \left( \frac{r}{\beta }+1,\left[ n+1\right] \left( t/\alpha \right) ^{\beta }\right) \end{aligned}$$

### Entropy measures

Entropy measures are important when highlighting a random variable’s uncertainty variation. Here, we present important entropy measures including the Reńyi entropy, Havrda and Charvat (HC) entropy, the Arimoto entropy and the Tsallis entropy based on the CBellW model. Moreover, we evaluate their numerical values which show flexibility under the CBellW model. For more details, the readers are referred to^12^. In the following let *X* $\sim$ CBellW ($\alpha ,\,\beta ,\,\lambda$).

The *Reńyi entropy* is given by

18 $$\begin{aligned} R_{\delta }=\left( 1-\delta \right) ^{-1}\log \left[ \sum _{k=0}^{\infty }Q_{k}\,\frac{\beta ^{\delta -1}\,\alpha ^{1-\delta }}{\left[ k+\delta \right] ^{\delta -\frac{\delta }{\beta }+\frac{1}{\beta }}}\Gamma \left( \delta -\frac{\delta }{\beta }+\frac{1}{\beta }\right) \right] , \end{aligned}$$

where $$Q_{k}=\sum _{b=0}^{\infty }Q_{b}\left( -1\right) ^{k}\left( {\begin{array}{c}b\\ k\end{array}}\right)$$ and

19 $$\begin{aligned} Q_{b}=\frac{\left( 1+\delta \right) ^{b}\lambda ^{\left( \delta +b\right) }}{\left[ \exp \left( e^{\lambda }-1\right) -1\right] ^{\delta }b!}\sum _{t,s}^{\infty }\left( -1\right) ^{\left( t+s\right) }\frac{\delta ^{t}}{t!}. \end{aligned}$$

The *HC entropy* is given by

20 $$\begin{aligned} HC_{\delta }=\frac{1}{2^{1-\delta }-1}\left[ \sum _{k=0}^{\infty }Q_{k}\,\frac{\beta ^{\delta -1}\,\alpha ^{1-\delta }}{\left[ k+\delta \right] ^{\delta -\frac{\delta }{\beta }+\frac{1}{\beta }}}\Gamma \left( \delta -\frac{\delta }{\beta }+\frac{1}{\beta }\right) -1\right] . \end{aligned}$$

The *Arimoto entropy* is given as follows:

21 $$\begin{aligned} A_{\delta }=\frac{\delta }{1-\delta }\left\{ \left[ \sum _{k=0}^{\infty }Q_{k}\,\frac{\beta ^{\delta -1}\,\alpha ^{1-\delta }}{\left[ k+\delta \right] ^{\delta -\frac{\delta }{\beta }+\frac{1}{\beta }}}\Gamma \left( \delta -\frac{\delta }{\beta }+\frac{1}{\beta }\right) \right] ^{\frac{1}{\delta }}-1\right\} \end{aligned}$$

The *Tsallis entropy* is given by

22 $$\begin{aligned} T_{\delta }=\frac{1}{\delta -1}\left[ 1-\sum _{k=0}^{\infty }Q_{k}\,\frac{\beta ^{\delta -1}\,\alpha ^{1-\delta }}{\left[ k+\delta \right] ^{\delta -\frac{\delta }{\beta }+\frac{1}{\beta }}}\Gamma \left( \delta -\frac{\delta }{\beta }+\frac{1}{\beta }\right) \right] . \end{aligned}$$

See Table 1 for exemplary numerical computations of the above entropy measures.

**Table 1:** Numerical computation of entropy measures.

$\lambda$	$\delta =0.7$		$\delta =2$			$\delta =0.7$		$\delta =2$	
	$\beta =0.8$	$\beta =6$	$\beta =0.8$	$\beta =6$	$\lambda$	$\beta =0.8$	$\beta =6$	$\beta =0.8$	$\beta =6$
Reńyi entropy					HC entropy				
0.5	− 1.7503	0.6616	− 0.9404	0.0887	0.5	2.7690	− 0.3426	1.2967	− 1.4280
1.0	− 2.3457	0.8755	− 1.0536	0.1365	1.0	3.3916	− 0.4777	1.5716	−2.0280
1.5	− 2.7523	1.2279	− 1.1521	0.2308	1.5	3.9389	− 0.7183	1.6841	− 3.1811
2.0	− 2.8974	1.6286	− 1.2107	0.3739	2.0	4.2341	− 1.0228	1.7119	2.0000
2.5	− 2.9444	− 49.7258	− 1.2328	0.5145	2.5	4.3341	− 1.2680	1.7212	2.0000
3.0	− 2.9828	− 85.6516	− 1.2445	0.6116	3.0	4.3977	− 4.3263	1.7288	1.9985
Tsallis entropy					Arimoto entropy				
0.5	− 2.1335	0.2640	− 0.6484	0.7140	0.5	− 0.9532	0.5123	− 3.1221	0.1697
1.0	− 2.6132	0.3680	− 0.7858	1.0140	1.0	− 1.1789	0.7009	− 3.7357	0.2552
1.5	− 3.0349	0.5534	− 0.8420	1.5905	1.5	− 1.3115	1.0393	− 4.3299	0.4122
2.0	− 3.2623	0.7880	− 0.8560	− 1.0000	2.0	− 1.3550	1.4699	− 4.7115	0.6239
2.5	− 3.3394	0.9770	− 0.8606	− 1.1104	2.5	− 1.3687	− 2.3333	− 4.8619	0.8043
3.0	− 3.3883	3.3333	− 0.8644	− 1.1002	3.0	− 1.3798	− 2.3333	− 4.9426	0.9150

### Parameter estimation

The log-likelihood function *L* related to the parameter vector $\theta =(\lambda ,\alpha ,\beta )^\top$ in Eq. (5) is given by

$$\begin{aligned} L= & {} n\log (\lambda )+n\log (\beta )-n\beta \log \left( \alpha \right) +(\beta -1)\sum _{i=1}^{n}\log x_{i}-\sum _{i=1}^{n}\left( x_{i}/\alpha \right) ^{\beta }\\{} & {} \quad +\lambda \sum _{i=1}^{n}\,\biggl (1-\exp \left[ -\left( x_{i}/\alpha \right) ^{\beta }\right] \biggr )+\sum _{i=1}^{n}\left\{ e^{\lambda \Bigl (1-\exp \left[ -\left( x_{i}/\alpha \right) ^{\beta }\right] \Bigr )}-1\right\} \\{} & {} \quad -n\log \left\{ \exp \left[ e^{\lambda }-1\right] -1\right\} . \end{aligned}$$

The components of the score vector $U(\theta )$ are as follows:

$$\begin{aligned} U_{\lambda }= & {} \frac{n}{\lambda }+\sum _{i=1}^{n}\,\biggl (1-\exp \left[ -\left( x_{i}/\alpha \right) ^{\beta }\right] \biggr )+\sum _{i=1}^{n}e^{\lambda \Bigl (1-\exp \left[ -\left( x_{i}/\alpha \right) ^{\beta }\right] \Bigr )}\Bigl (1-\exp \left[ -\left( x_{i}/\alpha \right) ^{\beta }\right] \Bigr )\\{} & {} -\frac{n\,e^{\lambda }\,\exp \left[ e^{\lambda }-1\right] }{\left\{ \exp \left[ e^{\lambda }-1\right] -1\right\} }, \\ U_{\alpha }= & {} -\frac{n\beta }{\alpha }+\frac{\beta }{\alpha ^{2}}\sum _{i=1}^{n}x_{i}\left( x_{i}/\alpha \right) ^{\beta -1}-\frac{\lambda \beta }{\alpha ^{2}}\sum _{i=1}^{n}x_{i}\exp \left[ -\left( x_{i}/\alpha \right) ^{\beta }\right] \,\left( x_{i}/\alpha \right) ^{\beta -1}\\{} & {} \quad -\sum _{i=1}^{n}\,\frac{\beta \lambda x_{i}\left( \frac{x_{i}}{\alpha }\right) {}^{\beta -1}e^{\lambda \left( 1-e^{-\left( \frac{x_{i}}{\alpha }\right) {}^{\beta }}\right) -\left( \frac{x_{i}}{\alpha }\right) {}^{\beta }}}{\alpha ^{2}}, \\ U_{\beta }= & {} \frac{n}{\beta }-n\log \left( \alpha \right) +\sum _{i=1}^{n}\log x_{i}-\sum _{i=1}^{n}\left( x_{i}/\alpha \right) ^{\beta }\log \left[ \frac{x_{i}}{a}\right] \\{} & {} \quad +\lambda \sum _{i=1}^{n}e^{-\left( \frac{x_{i}}{\alpha }\right) {}^{\beta }}\left( \frac{x_{i}}{\alpha }\right) {}^{\beta }\log \left( \frac{x_{i}}{\alpha }\right) +\sum _{i=1}^{n}\lambda \left( \frac{x_{i}}{\alpha }\right) {}^{\beta }\log \left( \frac{x_{i}}{\alpha }\right) e^{\lambda \left( 1-e^{-\left( \frac{x_{i}}{\alpha }\right) {}^{\beta }}\right) -\left( \frac{x_{i}}{\alpha }\right) {}^{\beta }}. \end{aligned}$$

By solving this system of non-linear equations, one can obtain the maximum likelihood estimates of the respective parameters. The above equations can be solved using computer-based programming algorithms.

## Simulation study

In this section, we conduct a simulation study related to the parameter estimates of the proposed CBellW model’s to analyze the performance for various sample sizes $n=20,25,30,\ldots ,250$. We simulated $N=1000$ samples that are replicated 5000 times. We consider the scale parameter $\alpha =2$ for two different sets and vary the shape parameters $\beta$ and $\lambda$ in various combinations. In particular, we consider $\text {set I}=[\beta = 6.0,\, \lambda = 0.70]$ and $\text {set II}=[\beta = 5.0,\,\lambda = 1.20]$.

**Figure 3:**
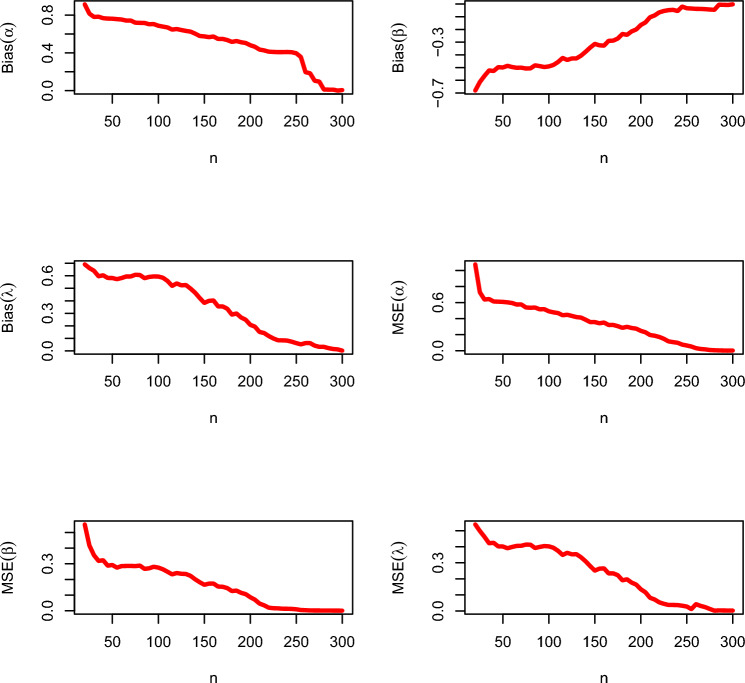
Graphical illustration of biases and MSEs for varying sample sizes for set I.

**Figure 4:**
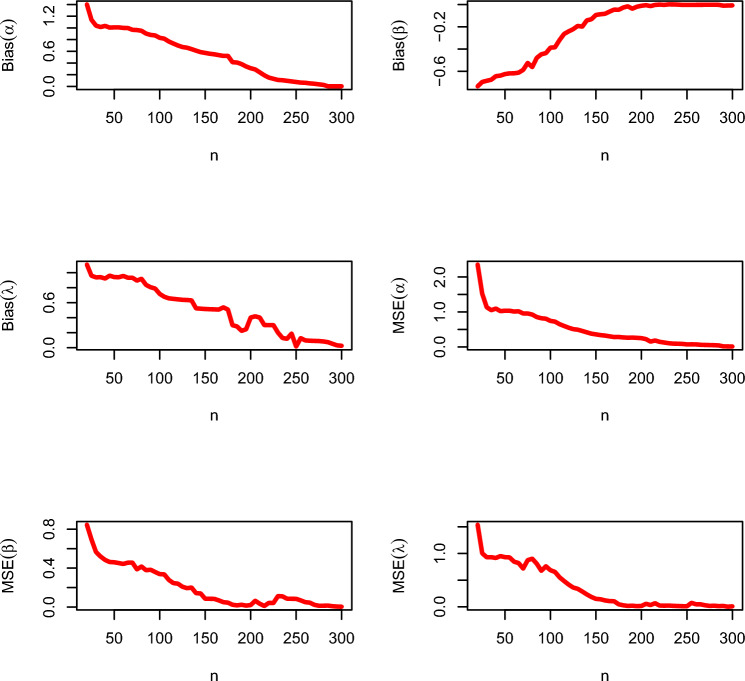
Graphical illustration of biases and MSEs for varying sample sizes for set II.

According to the results of the simulation study in Tables 2, 3, the bias and the mean squared error (MSE) of the parameters decrease as the sample size increases. Therefore, the CBellW model parameters may be estimated and their proposed confidence intervals can be constructed using the maximum likelihood estimators (MLEs) and their asymptotic results. The graphical illustration of MSEs and biases for set I and set II are presented in Figs. 3 and 4, respectively. The following Eqs. (23) and (24),

23 $$\begin{aligned} \text {MSE}(\hat{\Theta })=\sum _{r=1}^{5,000}\frac{(\hat{\Theta _{i}}-\Theta )^{2}}{5,000} \end{aligned}$$

and

24 $$\begin{aligned} \text {Bias}(\hat{\Theta })=\sum _{r=1}^{5,000}\frac{\hat{\Theta _{i}}}{5,000}-\Theta , \end{aligned}$$

are used to evaluate the MSE and bias of the estimates, respectively.

**Table 2:** Output summary of simulation study regarding set I.

*n*	Bias			MSE		
	$\alpha$	$\beta$	$\lambda$	$\alpha$	$\beta$	$\lambda$
20	0.91431	− 0.68093	0.69129	1.07612	0.55150	0.49831
30	0.78086	− 0.56554	0.64027	0.63843	0.35478	0.42194
40	0.76757	− 0.52670	0.60396	0.61330	0.32234	0.40203
50	0.76188	− 0.49932	0.58213	0.60866	0.29232	0.39065
60	0.75342	− 0.49564	0.58196	0.59432	0.28512	0.40531
70	0.74258	− 0.50062	0.59449	0.57701	0.28623	0.41355
80	0.71772	− 0.50628	0.60604	0.53503	0.28835	0.39222
90	0.70353	− 0.48847	0.59084	0.51608	0.27110	0.40433
100	0.68738	− 0.49102	0.59387	0.49110	0.27560	0.39379
110	0.66942	− 0.45618	0.55849	0.46901	0.24895	0.34887
115	0.64684	− 0.42436	0.51938	0.44176	0.23238	0.36222
120	0.65268	− 0.44012	0.53867	0.44771	0.24092	0.35269
125	0.64213	− 0.42827	0.52373	0.43390	0.23614	0.35376
130	0.63271	− 0.42809	0.52604	0.41899	0.23489	0.33447
135	0.62524	− 0.40622	0.49778	0.41286	0.22299	0.30935
140	0.60382	− 0.37564	0.46489	0.38588	0.20102	0.27918
145	0.57920	− 0.34240	0.42240	0.35641	0.18233	0.25138
150	0.57467	− 0.31360	0.38347	0.35687	0.16583	0.26367
155	0.56676	− 0.32498	0.39982	0.34173	0.17320	0.26507
160	0.57324	− 0.32729	0.40258	0.35107	0.17368	0.23401
165	0.54747	− 0.28960	0.35547	0.32049	0.15441	0.23433
170	0.54800	− 0.28800	0.35520	0.32113	0.15313	0.22291
175	0.53591	− 0.27129	0.33644	0.30592	0.14453	0.19115
180	0.51644	− 0.23556	0.28978	0.28504	0.12590	0.19707
185	0.52587	− 0.24213	0.29867	0.29762	0.12866	0.17677
190	0.51271	− 0.21529	0.26684	0.28317	0.11443	0.16422
195	0.50489	− 0.20018	0.24862	0.27479	0.10609	0.13493
200	0.48116	− 0.16498	0.20809	0.24738	0.08632	0.11646
205	0.46489	− 0.14498	0.19369	0.22978	0.07076	0.08219
210	0.43520	− 0.10373	0.15120	0.19579	0.04585	0.07145
215	0.42640	− 0.08987	0.14267	0.18656	0.03479	0.05382
220	0.41316	− 0.06578	0.11876	0.17158	0.01964	0.04444
225	0.41004	− 0.05502	0.09964	0.14860	0.01626	0.03755
230	0.40809	− 0.04844	0.08489	0.11683	0.01539	0.03731
235	0.40818	− 0.04569	0.08418	0.10691	0.01293	0.03652
240	0.40942	− 0.05498	0.08249	0.09890	0.01274	0.03256
245	0.40711	− 0.02036	0.07351	0.07600	0.01174	0.02750
250	0.39604	− 0.03342	0.06204	0.06510	0.00928	0.01165
255	0.35601	− 0.03542	0.05204	0.05169	0.00459	0.04219
260	0.19608	− 0.03812	0.06204	0.03167	0.00348	0.03145
265	0.18403	− 0.03842	0.06204	0.02167	0.00196	0.02382
270	0.10502	− 0.04042	0.04204	0.01689	0.00163	0.01245
275	0.09600	− 0.04342	0.03204	0.00866	0.00154	0.00176
280	0.01240	− 0.04534	0.03204	0.00565	0.00129	0.00327
285	0.01100	− 0.00534	0.02204	0.00365	0.00127	0.00287
290	0.01092	− 0.00634	0.01520	0.00225	0.00092	0.00253
295	0.00100	− 0.00734	0.01204	0.00120	0.00073	0.00208
300	0.00600	− 0.00234	0.00200	0.00110	0.00052	0.00206

**Table 3:** Output summary of simulation study regarding set II.

*n*	Bias			MSE		
	$\alpha$	$\beta$	$\lambda$	$\alpha$	$\beta$	$\lambda$
20	1.40181	− 0.73479	1.10748	2.35450	0.84589	1.53864
30	1.03838	− 0.68495	0.93694	1.13224	0.56531	0.92927
40	1.03407	− 0.64343	0.92194	1.09596	0.48425	0.91634
50	1.00898	− 0.62477	0.94097	1.03486	0.46007	0.93100
60	0.99972	− 0.61778	0.95597	1.01184	0.44309	0.84672
70	0.96736	− 0.58722	0.93236	0.95550	0.45654	0.71726
80	0.95069	− 0.56181	0.91778	0.92485	0.41652	0.90210
90	0.87986	− 0.44639	0.80569	0.81958	0.38312	0.67620
100	0.83042	− 0.38708	0.71667	0.74326	0.33826	0.68639
110	0.76750	− 0.31875	0.65775	0.64958	0.27896	0.55458
115	0.73264	− 0.26361	0.65093	0.59829	0.24600	0.48348
120	0.69931	− 0.24069	0.64435	0.54802	0.24010	0.41695
125	0.67292	− 0.22083	0.63804	0.50785	0.20847	0.36160
130	0.66097	− 0.19153	0.63542	0.49149	0.19357	0.33778
135	0.63708	− 0.19792	0.63008	0.45507	0.20007	0.28694
140	0.61083	− 0.14167	0.52446	0.41611	0.14236	0.23299
145	0.58583	− 0.13167	0.51996	0.37819	0.13944	0.18507
150	0.57208	− 0.09292	0.51558	0.35757	0.08507	0.14944
155	0.55833	− 0.08667	0.51258	0.33694	0.08444	0.14207
160	0.54875	− 0.08250	0.51075	0.32313	0.08375	0.12025
165	0.53458	− 0.06292	0.50796	0.30174	0.06799	0.10742
170	0.52125	− 0.04500	0.53750	0.28146	0.04958	0.10477
175	0.52208	− 0.04667	0.50833	0.28299	0.04486	0.04736
180	0.41375	− 0.02625	0.30120	0.27063	0.02313	0.02875
185	0.40833	− 0.01542	0.28333	0.26236	0.01674	0.01736
190	0.38102	− 0.02375	0.22510	0.26505	0.02313	0.02125
195	0.34058	− 0.01292	0.24583	0.25861	0.01424	0.01299
200	0.31067	− 0.01917	0.40192	0.24986	0.01986	0.01611
205	0.29017	− 0.01042	0.41817	0.22194	0.06403	0.05694
210	0.24013	− 0.00500	0.40150	0.15146	0.03333	0.03333
215	0.19021	− 0.01333	0.30148	0.18243	0.01056	0.06806
220	0.15004	− 0.00292	0.30124	0.14505	0.04236	0.02361
225	0.13041	− 0.02141	0.30125	0.12502	0.04114	0.02114
230	0.11130	− 0.00250	0.20105	0.10505	0.11236	0.02330
235	0.10505	0.00201	0.12911	0.09250	0.10944	0.01851
240	0.09524	0.00150	0.11920	0.09025	0.08507	0.01494
245	0.08518	− 0.00134	0.18802	0.08525	0.08444	0.01207
250	0.07542	− 0.00250	0.01583	0.07125	0.08375	0.01025
255	0.06504	− 0.00235	0.12753	0.07325	0.06799	0.07424
260	0.06150	− 0.00210	0.09710	0.07125	0.04958	0.04771
265	0.05151	− 0.00190	0.09269	0.05925	0.04486	0.04736
270	0.04514	− 0.00180	0.09058	0.05525	0.02213	0.02875
275	0.03524	− 0.00140	0.08852	0.05125	0.01274	0.01736
280	0.02551	− 0.00120	0.08340	0.04825	0.01313	0.02125
285	0.00215	− 0.00110	0.07335	0.04225	0.01424	0.01299
290	0.00195	− 0.00180	0.05180	0.01525	0.00815	0.01611
295	0.00105	− 0.00980	0.03102	0.01253	0.00450	0.00236
300	0.00253	− 0.01535	0.02580	0.01053	0.00312	0.00890

## Real-life applications

This section aims to practically implement the CBellW model on real data sets to demonstrate the benefits of the proposed model. In “Modeling of COVID-19 and cancer data” section, we apply the CBellW model to four medical data sets, and in the following “Designing a GASP with application to Guinea pigs data” and “Actuarial measures with applications to auto-mobile collision claims data” section, we design a GASP (with application to Guinea pigs data) and compute risk measures by using actuarial data, respectively. We also compare several Weibull-based models such as the complementary Poisson Weibull (CPW)^3^, alpha power Weibull (APW)^13^, transmuted Weibull (TW)^14^, beta Weibull (BW)^15^, Marshall Olkin Weibull (MOW)^16^, Weibull claim (W-claim)^17^, gamma Weibull (GW)^18^, Gull alpha power Weibull (GAPW)^19^, and exponentiated exponential (EE) with the proposed CBellW model.

The first data set was recently used by^20^ and comprises daily confirmed COVID-19 death cases. The data set consists of 89 observations with an average of 18.72 daily reported deaths. The second data set represents the survival time of head and neck cancer disease patients treated by using radiotherapy (RT). The data set consists of 58 observations with a mean survival time of 226.17. This data set is also used by^21^. The third data set contains 128 people with blood cancer’s average number of months in remission with a mean remission time of 9.37 months and was recently examined by many authors including Hamdeni et al.^22^ and^23^. The fourth data set was recently used by^23^ and represents the survival times in days of 73 patients diagnosed with acute bone cancer with mean survival time of 3.76. The fifth data set^24^ represents the survival data of Guinea pigs infected with virulent tubercle bacilli. Guinea pigs are regarded to have a high susceptibility to human tuberculosis, which is one of the motives to select guinea pigs for this study. The sixth data set is extracted from the Insurance Data R package^25^ and represents UK auto-mobile collision claims. The data set consists of 32 observations (in pounds) related to the severity of claims. The observations are divided by 100 for computational purposes (but this does not affect statistical inference). The descriptive statistics for all the data sets are shown in Table 4, whereas the data sets 1–5 are given in Table 5 (for data set 6 see^25^).

**Table 4:** Descriptive information on the data sets.

	*n*	$x_0$	$Q_1$	$\widetilde{x}$	$\bar{x}$	$Q_3$	$x_n$	$\sigma$	$S_k$	*K*
Data-1	89	1.00	8.00	14.00	18.72	21.00	97.00	17.20	2.24	9.57
Data-2	58	6.53	83.25	151.50	226.17	237.00	1417.0	273.94	2.72	10.72
Data-3	128	0.08	3.35	6.40	9.37	11.84	79.05	10.51	3.29	18.48
Data-4	73	0.09	0.92	1.57	3.76	2.75	86.01	10.60	6.80	51.78
Data-5	72	0.10	1.08	1.50	1.77	2.24	5.55	1.03	1.34	4.99
Data-6	32	1.54	2.12	2.51	2.76	2.98	7.98	1.10	3.38	16.70

**Table 5:** Real data sets.

Data-1: COVID-19									
1.00	1.00	2.00	4.00	5.00	1.00	1.00	3.00	6.00	6.00
4.00	1.00	5.00	6.00	6.00	8.00	5.00	7.00	7.00	9.00
9.00	15.00	17.00	11.00	13.00	5.00	14.00	5.00	13.00	9.00
19.00	15.00	11.00	14.00	12.00	11.00	7.00	13.00	10.00	20.00
22.00	21.00	12.00	14.00	9.00	14.00	7.00	16.00	17.00	13.00
21.00	11.00	11.00	8.00	11.00	12.00	15.00	21.00	20.00	18.00
15.00	14.00	21.00	16.00	11.00	28.00	29.00	19.00	14.00	19.00
29.00	34.00	34.00	46.00	46.00	47.00	36.00	38.00	40.00	32.00
39.00	34.00	35.00	36.00	35.00	45.00	62.00	91.00	97.00	
Data-2: Head & Neck Cancer									
6.53	7.00	10.42	14.48	16.10	22.70	34.00	41.55	42.00	45.28
49.40	53.62	63.00	64.00	83.00	84.00	91.00	108.00	112.00	129.00
133.00	133.00	139.00	140.00	140.00	146.00	149.00	154.00	157.00	160.00
160.00	165.00	146.00	149.00	154.00	157.00	160.00	160.00	165.00	173.00
176.00	218.00	225.00	241.00	248.00	273.00	277.00	297.00	405.00	417.00
420.00	440.00	523.00	583.00	594.00	1101.00	1146.00	1417.00		
Data-3: Bladder Cancer									
0.080	2.090	3.480	4.870	6.940	8.660	13.110	23.630	0.200	2.230
3.520	4.980	6.970	9.020	13.290	0.400	2.260	3.570	5.060	7.090
9.220	13.80	25.74	0.500	2.460	3.640	5.090	7.260	9.470	14.24
25.82	0.510	2.540	3.700	5.170	7.280	9.740	14.76	26.31	0.810
2.620	3.820	5.320	7.320	10.060	14.77	32.15	2.640	3.880	5.320
7.390	10.34	14.830	34.26	0.900	2.690	4.180	5.340	7.590	10.66
15.96	36.66	1.050	2.690	4.230	5.410	7.620	10.75	16.62	43.010
1.190	2.750	4.260	5.410	7.630	17.12	46.12	1.260	2.830	4.330
5.490	7.660	11.250	17.14	79.050	1.350	2.870	5.620	7.870	11.64
17.360	1.400	3.020	4.340	5.710	7.930	11.79	18.100	1.460	4.400
5.850	8.260	11.980	19.130	1.760	3.250	4.500	6.250	8.370	12.02
2.020	3.310	4.510	6.540	8.530	12.030	20.28	2.020	3.360	6.760
12.07	21.73	2.070	3.360	6.930	8.650	12.63	22.690		
Data-4: Acute Bone Cancer									
0.090	0.760	1.810	1.100	3.720	0.720	2.490	1.000	0.530	0.660
31.610	0.600	0.200	1.610	1.880	0.700	1.360	0.430	3.160	1.570
4.930	11.070	1.630	1.390	4.540	3.120	86.010	1.920	0.920	4.040
1.160	2.260	0.200	0.940	1.820	3.990	1.460	2.750	1.380	2.760
1.860	2.680	1.760	0.670	1.290	1.560	2.830	0.710	1.480	2.410
0.660	0.650	2.360	1.290	13.750	0.670	3.700	0.760	3.630	0.680
2.650	0.950	2.300	2.570	0.610	3.930	1.560	1.290	9.940	1.670
1.420	4.180	1.370							
Data-5: Guinea Pigs									
0.100	0.330	0.440	0.560	0.590	0.720	0.740	0.770	0.920	0.930
0.960	1.000	1.000	1.020	1.050	1.070	1.070	1.080	1.080	1.080
1.090	1.120	1.130	1.150	1.160	1.200	1.210	1.220	1.220	1.240
1.300	1.340	1.360	1.390	1.440	1.460	1.530	1.590	1.600	1.630
1.630	1.680	1.710	1.720	1.760	1.830	1.950	1.960	1.970	2.020
2.130	2.150	2.160	2.220	2.300	2.310	2.400	2.450	2.510	2.530
2.540	2.540	2.780	2.930	3.270	3.420	3.470	3.610	4.020	4.320
4.580	5.550								

### Modeling of COVID-19 and cancer data

From a medical perspective, policy makers are always interested in accurate estimates to enable better planning for disease management and control. There are several flexible models that are commonly used for this purpose, e.g., Klakattawi et al.^23^ used an extended Weibull model for cancer patients survival analysis. Badr et al.^26^ employed an extended Weibull distribution on survival data. Zichuan et al.^27^ analysed bladder cancer data also by using an extended Weibull distribution, and Wang et al.^28^ introduced an exponent power Weibull model to analyze medical data.

**Figure 5:**
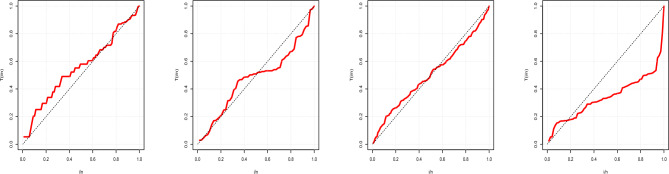
TTT plots of Data-1 – Data-4.

**Figure 6:**
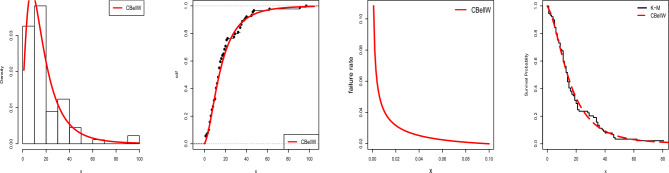
Estimated pdf, cdf, hrf, and survival function for Data-1.

**Figure 7:**
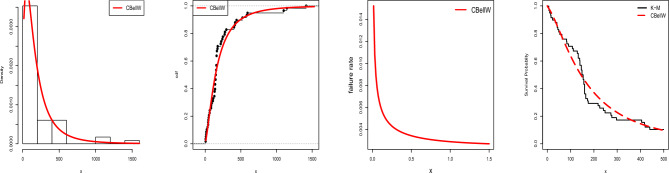
Estimated pdf, cdf, hrf, and survival function for Data-2.

**Figure 8:**
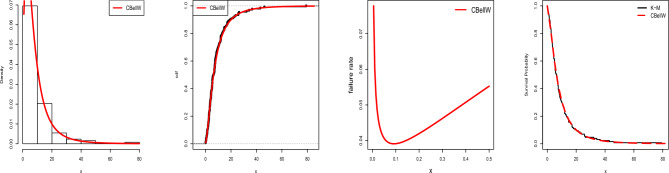
Estimated pdf, cdf, hrf, and survival function for Data-3.

**Figure 9:**
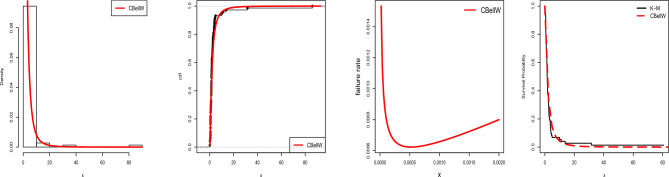
Estimated pdf, cdf, hrf, and survival function for Data-4.

**Figure 10:**
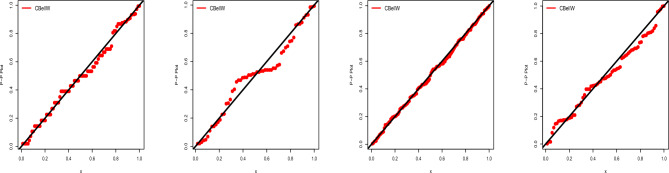
Probability–Probability (P–P) plot of Data-1-4.

In the following, we focus on data sets 1–4 (COVID-19 and cancer data). Table 6 displays the MLEs and SEs of the estimates for the fitted models. AIC, CAIC, BIC, and HQIC are shown in Table 7 along with other important metrics like *p*-values and the results of the Anderson-Darling (A), Cramer-von Mises (W), and Kolmogrov–Smirnov (K–S) tests. See also some related visualizations in Figs. 5, 6, 7, 8, 9 and 10. Following the results it can be stated that the proposed CBellW model with three parameters outperforms the other well-known models. Among all other comparable models, the model with the highest *p*-values and lowest values of the information criteria is deemed to be the best.

**Table 6:** Fitted models with parameter estimates and standard errors.

Dist.	Par.	Data-1		Data-2		Data-3		Data-4	
		Est.	SE	Est.	SE	Est.	SE	Est.	SE
CBellW	$\hat{\alpha }$	2.158	1.5998	12.307	13.954	0.5276	0.3668	0.0100	0.0063
	$\hat{\beta }$	0.5320	0.1108	0.4222	0.1068	0.4350	0.0684	0.2823	0.0243
	$\hat{\lambda }$	1.8005	0.3090	1.8773	0.3753	1.9526	0.2450	2.8434	0.2404
CPW	$\hat{\alpha }$	0.0708	0.0094	0.0053	0.0026	0.0041	0.0009	0.0146	0.0129
	$\hat{\beta }$	0.0803	0.1067	0.1722	0.0083	0.2191	0.0071	0.2893	0.0338
	$\hat{\lambda }$	20.085	24.568	191.73	68.56	87.518	16.448	35.257	15.1860
APW	$\hat{\alpha }$	0.0048	0.0014	0.0186	0.0293	0.0168	0.0069	1.5214	0.1597
	$\hat{\beta }$	1.3023	0.1283	0.8113	0.2063	1.2670	0.0859	0.4928	0.0433
	$\hat{\gamma }$	0.0063	0.0134	4.1631	8.6022	0.0156	0.0248	150.485	99.297
TW	$\hat{\alpha }$	0.0134	0.0048	0.0035	0.0010	0.3026	0.0617	0.2337	0.0398
	$\hat{\beta }$	1.2863	0.1004	0.9859	0.0582	0.7542	0.0686	0.8583	0.0627
	$\hat{\lambda }$	0.7183	0.2857	0.5467	0.4318	1.0103	0.1112	0.8557	0.1110
W	$\hat{\alpha }$	0.0281	0.0093	0.0062	0.0025	0.0939	0.0191	0.4395	0.0687
	$\hat{\beta }$	1.1936	0.0932	0.9443	0.0677	1.0478	0.0675	0.7656	0.0568
EE	$\hat{\alpha }$	0.0683	0.0087	0.0047	0.0008	0.1212	0.0136	0.2229	0.0397
	$\hat{\beta }$	1.4932	0.2253	1.0835	0.1889	1.2180	0.1488	0.7887	0.1259
BW	$\hat{\alpha }$	0.1652	0.1989	0.0845	0.1105	0.4732	0.3641	3.7522	1.0982
	$\hat{\beta }$	0.8327	0.3824	0.5969	0.3481	0.6685	0.2418	0.2401	0.1087
	$\hat{a}$	2.1565	1.5026	2.9468	2.8047	2.7231	1.5721	58.1811	70.9110
	$\hat{b}$	0.8347	2.1504	0.9433	2.1961	0.8903	1.4496	1.1762	0.9420
W-Claim	$\hat{\alpha }$	0.1973	0.2389	0.7261	0.6214	0.3832	0.2834	5.0850	0.3323
	$\hat{\beta }$	0.7749	0.2219	0.3580	0.1025	0.7090	0.1492	0.2670	0.0262
	$\hat{\lambda }$	0.4776	0.9624	0.0135	0.0215	0.5881	0.8854	0.0030	0.0008
MOW	$\hat{\alpha }$	1.5491	0.7813	0.0058	0.0033	3.1310	0.8477	3.9605	0.5737
	$\hat{\beta }$	0.4088	0.0962	0.9525	0.0735	0.3078	0.0533	0.3141	0.0390
	$\hat{a}$	97.112	113.01	0.9829	0.4702	256.31	247.93	99.99	58.44
GW	$\hat{\alpha }$	0.3957	0.6711	0.0040	0.0006	1.3087	1.4908	9.4447	0.0538
	$\hat{\beta }$	0.6871	0.2977	7.5774	0.2290	0.5203	0.1951	0.2692	0.0189
	$\hat{\lambda }$	2.7489	2.1652	0.1358	0.0240	3.7459	2.6219	11.3826	0.4015
GAPW	$\hat{\alpha }$	0.0095	0.0036	0.1856	0.1203	0.0346	0.0091	0.1752	0.0316
	$\hat{\beta }$	1.3351	0.1011	0.4962	0.0953	1.1834	0.0783	0.9077	0.0654
	$\hat{\gamma }$	2.2099	0.5194	0.0062	0.0142	2.2909	0.3543	2.4789	0.1856

**Table 7:** Detailed summary of model selection criteria.

Dist.	LL	AIC	CAIC	BIC	HQIC	A	W	K-S	*p*-value
Data-1: COVID-19									
CBellW	345.853	697.705	697.988	705.171	700.715	0.4356	0.0655	0.0646	0.8511
CPW	346.593	699.185	699.468	706.651	702.195	0.6598	0.1120	0.0887	0.4863
APW	346.214	698.428	698.710	705.894	701.437	0.5366	0.0827	0.0842	0.5541
TW	346.383	698.767	699.049	706.233	701.776	0.6540	0.1108	0.0845	0.5487
W	347.431	698.863	699.002	703.840	700.869	0.7886	0.1374	0.0976	0.3642
EE	346.394	699.428	699.010	706.894	702.437	0.6485	0.1065	0.0863	0.5209
BW	346.047	700.094	700.571	710.049	704.107	0.5971	0.0891	0.0739	0.7158
W-Claim	346.002	698.004	698.287	705.470	701.013	0.5859	0.0926	0.0768	0.6696
MOW	346.779	699.559	699.841	707.025	702.568	0.6091	0.1040	0.0702	0.7732
GW	346.110	698.220	698.502	705.686	701.229	0.6120	0.0917	0.0750	0.6992
GAPW	346.104	698.209	698.491	705.675	701.218	0.6041	0.0993	0.0791	0.6343
Data-2: Head & Neck Cancer									
CBellW	370.564	747.128	747.572	753.309	749.536	0.8790	0.1833	0.1299	0.2814
CPW	375.322	756.644	757.089	762.825	759.052	1.9813	0.3822	0.1968	0.0224
APW	372.438	750.875	751.320	757.056	753.283	1.2351	0.2429	0.1544	0.1259
TW	371.686	749.371	749.816	755.552	751.779	1.0646	0.2160	0.1419	0.1936
W	372.444	748.888	749.106	753.009	750.493	1.2151	0.2410	0.1503	0.1455
EE	372.390	748.779	748.997	752.900	750.384	1.2526	0.2470	0.1680	0.0758
BW	370.771	749.542	750.297	757.784	752.753	1.0208	0.2118	0.1378	0.2210
W-Claim	370.563	747.127	747.571	753.308	749.534	0.8861	0.1805	0.1348	0.2429
MOW	372.414	750.827	751.272	757.009	753.235	1.2161	0.2411	0.1518	0.1382
GW	373.203	752.407	752.851	758.588	754.814	1.3748	0.2685	0.1787	0.0492
GAPW	370.618	747.235	747.680	753.417	749.643	0.9310	0.1940	0.1319	0.2651
Data-3: Bladder Cancer									
CBellW	409.596	825.192	825.385	833.748	828.668	0.1038	0.0163	0.0302	0.9998
CPW	420.832	847.664	847.857	856.220	851.140	1.6327	0.2512	0.0784	0.4117
APW	410.191	826.382	826.576	834.938	829.859	0.2539	0.0422	0.0465	0.9444
TW	410.978	827.956	828.149	836.512	831.432	0.3515	0.0556	0.0498	0.9088
W	414.087	832.174	832.270	837.878	834.491	0.7864	0.1314	0.0700	0.5574
EE	413.078	830.155	830.251	835.859	832.473	0.6741	0.1122	0.0725	0.5115
BW	410.679	829.357	829.683	840.765	833.993	0.2882	0.0436	0.0449	0.9583
W-Claim	410.917	827.834	828.028	836.391	831.311	0.3480	0.0563	0.0500	0.9056
MOW	410.866	827.733	827.926	836.289	831.209	0.3089	0.0533	0.0478	0.9322
GW	410.854	827.708	827.902	836.264	831.185	0.3143	0.0479	0.0468	0.9416
GAPW	411.344	828.687	828.881	837.243	832.163	0.4237	0.0708	0.0548	0.8371
Data-4: Acute Bone Cancer									
CBellW	142.831	291.662	292.010	298.534	294.401	0.9736	0.1348	0.0854	0.6613
CPW	143.145	292.290	292.638	299.162	295.029	1.0347	0.1443	0.0919	0.5687
APW	152.079	310.158	310.506	317.029	312.896	2.2840	0.3529	0.1493	0.0772
TW	156.614	319.228	319.576	326.100	321.967	2.9863	0.4809	0.1744	0.0235
W	161.402	326.803	326.975	331.384	328.629	3.6452	0.6014	0.1887	0.0111
EE	168.452	340.904	341.075	345.485	342.729	5.0318	0.8620	0.2316	0.0008
BW	143.039	294.077	294.665	303.239	297.728	1.0265	0.1429	0.0910	0.5813
W-Claim	147.567	301.134	301.481	308.005	303.872	1.6081	0.2361	0.1297	0.1714
MOW	150.095	306.189	306.537	313.061	308.928	1.9460	0.2922	0.1514	0.0706
GW	147.756	301.512	301.860	308.383	304.250	1.7260	0.2597	0.1190	0.2523
GAPW	155.277	316.554	316.902	323.425	319.292	2.7779	0.4435	0.1667	0.0345

### Designing a GASP with application to Guinea pigs data

Product quality is one of the most important characteristics that distinguish different goods in a global market. Before approving or rejecting a lot, particular quality control procedures are carried out in accordance with different sample schemes. A lot of items will be accepted or rejected in accordance with the acceptance sampling technique depending on the quality of the items that were assessed in a sample taken from the lot^29^. The GASP inspects multiple items at once depending on the number of testers available to the experimenter for testing, whereas the ordinary acceptance sampling plan (OASP) only inspects one item at a time.

This section provides an example of a GASP having cdf as in Eq. (26) with known parameters $\beta$ and $\lambda$ to demonstrate the assumption that an item’s lifespan distribution will follow the CBellW model. A sample of size *n* should be collected for a GASP, distributed, and retained for life testing for a predetermined period of time, where $n=rg$ with *r* items for each group. If any group experiences more failures than the acceptance number *c*, the experiment is declared a failure. Many authors have briefly described GASPs, and it can be found in, e.g.,^30–34^. When designing the GASP, the quality parameter is taken into consideration as either the mean or the median; however, for skewed distributions, the median is typically preferred^30^. The GASP is based on the following steps:

- Identify the group size *g*.
- Assign *r* items to each group for the life test after selecting *gr* items at random from a lot; in the life test, $n= gr$ is the necessary sample size.
- Set the life test’s termination time $t_0$ and the acceptance number *c* for each group.
- A decision is finally made to either accept or reject the lot. A lot can be accepted when there is a maximum of *c* nonconforming units, and it is to be rejected when there are more than *c* nonconforming units.

The probability of accepting a lot is given as follows:

25 $$\begin{aligned} p_{a\left( p\right) }=\left[ \sum _{i=0}^{c}\left( {\begin{array}{c}r\\ i\end{array}}\right) p^{i}\left[ 1-p\right] ^{r-i}\right] ^{g}, \end{aligned}$$

where *p* is used to signify the likelihood that a group member would fail before $t_0$ and is produced by inserting Eq. (7) in Eq. (4):

26 $$\begin{aligned} m=\alpha \left[ -\log \left( 1-\left\{ \lambda ^{-1}\,\log \left[ 1+\log \left\{ 1+p[\exp (e^{\lambda }-1)-1]\right\} \right] \right\} \right) \right] ^{1/\beta }, \end{aligned}$$

In the following, let

$$\begin{aligned} \zeta =\left[ -\log \left( 1-\left\{ \lambda ^{-1}\,\log \left[ 1+\log \left\{ 1+p[\exp (e^{\lambda }-1)-1]\right\} \right] \right\} \right) \right] ^{1/\beta }. \end{aligned}$$

By replacing $\alpha =m/\zeta$ and $t=a_{1}m_{0}$ in Eq. (4), we obtain the probability of a failure as

27 $$\begin{aligned} F_{\text {CBellW}}(t)=\frac{\exp \left[ e^{\lambda \left\{ 1-\exp \left[ -\left( \frac{a_{1}\zeta }{r_{2}}\right) ^{\beta }\right] \right\} }-1\right] -1}{\exp \left[ e^{\lambda }-1\right] -1}. \end{aligned}$$

Given $a_1$ and $r_2$, where $r_2=m/m_0$, *p* may be calculated for a chosen $\beta$ and $\lambda$ from Eq. (27). Both failure probabilities, which correspond to the consumer’s and producer’s risk, are denoted by $p_1$ and $p_2$, respectively. We have to determine the values of the design parameters (*c*, *g*) that concurrently meet both of the following equations for a given value of $\theta$ and $\lambda$, $r_2$, $a_1$, $\beta$, and $\gamma$

28 $$\begin{aligned} p_{a\left( p_{1}|\frac{m}{m_{0}}=r_{1}\right) }=\left[ \sum _{i=0}^{c}\left( {\begin{array}{c}r\\ i\end{array}}\right) p_{1}^{i}\left[ 1-p_{1}\right] ^{r-i}\right] ^{g}\le \beta , \end{aligned}$$

and

29 $$\begin{aligned} p_{a\left( p_{2}|\frac{m}{m_{0}}=r_{2}\right) }=\left[ \sum _{i=0}^{c}\left( {\begin{array}{c}r\\ i\end{array}}\right) p_{2}^{i}\left[ 1-p_{2}\right] ^{r-i}\right] ^{g}\ge 1-\gamma , \end{aligned}$$

where $r_1$ and $r_2$ represent the mean ratio at producer’s risk and consumer’s risk, respectively, and the failure probabilities to be used in Eqs. (28) and (29) are given in the following Eqs. (30) and (31) for the CBellW model:

30 $$\begin{aligned} p_{1}=\frac{\exp \left[ e^{\lambda \left\{ 1-\exp \left[ -\left( a_{1}\zeta \right) ^{\beta }\right] \right\} }-1\right] -1}{\exp \left[ e^{\lambda }-1\right] -1} \end{aligned}$$

and

31 $$\begin{aligned} p_{2}=\frac{\exp \left[ e^{\lambda \left\{ 1-\exp \left[ -\left( \frac{a_{1}\zeta }{r_{2}}\right) ^{\beta }\right] \right\} }-1\right] -1}{\exp \left[ e^{\lambda }-1\right] -1}. \end{aligned}$$

Table 8 shows the design parameters, which are obtained by taking $\beta =0.7330$ and $\lambda =2.0201$ and two levels of *r* (5, 10). The analysis revealed that by reducing $\beta$ (consumer’s risk) the number of groups tends to be increased. Moreover, the number of groups rapidly declines when $r_2$ increases. However, after a certain point, the probability of accepting a lot is increased with constant values of *g* and *c*. Table 8, where $\beta =0.25$, $a_1=1$, $\lambda =2.0201$ and $r=10$, indicating that *g* decreases and the OC value increases, shows the proposed GASP (see also Table 9).

**Figure 11:**
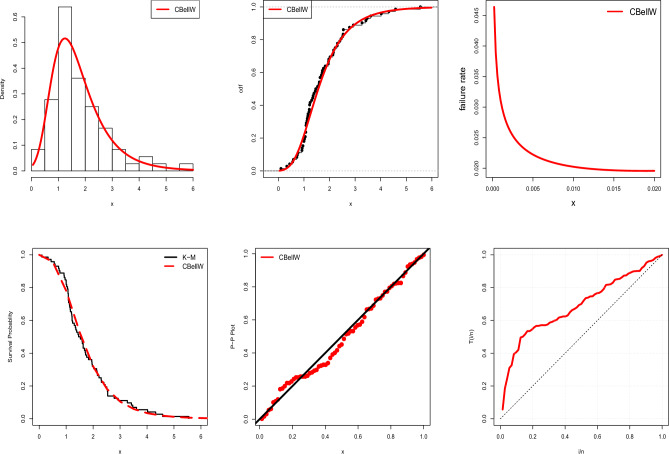
TTT plot and estimated pdf, cdf, hrf, K–M and P–P plot for Data-5.

Recently, Sivakumar et al.^24^ designed a GASP under the odd generalized exponential log-logistic model by analyzing survival data from guinea pigs that had been exposed to virulent tubercle bacilli. One of the factors that led researchers to choose guinea pigs for this investigation was their reputation for having a high vulnerability to human tuberculosis. Here, we bear in mind only the observations in which all animals in a single cage are below the identical regime. The data was also studied by Bjerkedal^35^. The data set consists of 72 observations of survival time with mean and median values of 1.77 and 1.51 days, respectively. See Fig. 11 for visualizations of the related data set. The K–S test led to a *p*-value of 0.617 and a maximum difference between real and fitted data of 0.089. In comparison to the odd generalized exponential log logistic model^24^, the three parameter CBellW model fits the data better (K–S test 0.0774 and *p*-value 0.7809). The estimated parameters (SEs) are $\hat{\alpha }=0.3418$ (0.1661), $\hat{\beta }=0.7330$ (0.1351) and $\hat{\lambda }=2.0201$ (0.2990). Table 8 shows the GASP under the CBellW model with MLE values suggesting minimal *g* and *c* for $r=5$ and $r=10$ and $a_1=0.5$ and 1, for lifetime testing. There are 90 groups, or 450 $(=90 \cdot 5)$ total units, required for testing. The number of groups or units that must be tested under identical conditions, however, is significantly reduced when $r=10$. As a result, a total of 12 groups or 120 $(=12\cdot 10)$ items is required for life testing. Here, a group size of 10 is preferred. Under the CBellW model, as the true median life grows, the number of groups reduces and the OC values rise.

**Table 8:** GASP under the CBellW model, $\beta =0.7330$ and $\lambda =2.0201$.

$\beta$	$r_2$	$r=5$						$r=10$					
		$a_1=0.5$			$a_1=1$			$a_1=0.5$			$a_1=1$		
		*g*	*c*	*p*(*a*)	*g*	*c*	*p*(*a*)	*g*	*c*	*p*(*a*)	*g*	*c*	*p*(*a*)
0.25	2	90	2	0.9885	3	2	0.9545	12	2	0.9832	3	1	0.9738
	4	12	1	0.9955	1	1	0.9946	4	1	0.9933	1	1	0.9777
	6	3	0	0.9516	1	1	0.9990	4	1	0.9981	1	1	0.9955
	8	3	0	0.9660	1	0	0.9694	2	0	0.9550	1	1	0.9983
0.1	2	149	2	0.9810	12	3	0.9876	19	2	0.9736	3	4	0.9875
	4	19	1	0.9928	2	1	0.9893	6	1	0.9900	1	1	0.9777
	6	19	1	0.9979	2	1	0.9979	6	1	0.9971	1	1	0.9955
	8	4	0	0.9550	1	0	0.9694	2	0	0.9550	1	1	0.9983
0.05	2	193	2	0.9755	15	3	0.9845	25	2	0.9653	4	4	0.9834
	4	24	1	0.9909	2	1	0.9893	7	1	0.9883	1	1	0.9777
	6	24	1	0.9974	2	1	0.9979	7	1	0.9966	1	1	0.9955
	8	24	1	0.9987	1	0	0.9694	7	1	0.9984	1	1	0.9983
0.01	2	297	2	0.9626	23	3	0.9764	174	3	0.9898	5	4	0.9793
	4	37	1	0.9860	3	1	0.9840	11	1	0.9817	2	1	0.9560
	6	37	1	0.9960	3	1	0.9969	11	1	0.9947	2	1	0.9910
	8	37	1	0.9981	3	1	0.9989	11	1	0.9974	2	1	0.9967

**Table 9:** Proposed GASP.

$r_2$	2	4	6	8
*n*	30	10	10	10
*g*	3	1	1	1
*c*	1	1	1	1
*p*(*a*)	0.9738	0.9777	0.9955	0.9983

### Actuarial measures with applications to auto-mobile collision claims data

Due to their adaptability and potential for precise predictions, extended models have become popular for investigating actuarial data. Many authors have used these models and emphasized their advantages^36–41^. As for the proposed CBellW model, we discuss different risk measures in the following.

The *Value at Risk* (VaR) is a statistical measure used in finance and risk management to estimate the potential losses on a financial portfolio or investment over a specified time horizon and at a given confidence level *q*. It quantifies the maximum loss that an investment or portfolio is expected to suffer under normal market conditions over a defined time frame. If a random variable *X* follows the CBellW distribution, then the following expression defines its VaR:

32 $$\begin{aligned} \text {VaR}_{q}=\alpha \Biggl (-\log \left[ 1-\Biggl \{\lambda ^{-1}\,\log \left[ 1+\log \left\{ 1+q[\exp (e^{\lambda }-1)-1]\right\} \right] \Biggr \}\right] \Biggr )^{1/\beta } \end{aligned}$$

The *Expected Shortfall* (ES), developed by Artzner et al.^42^ and typically regarded as superior to VaR, is another important financial indicator. It can be computed by

33 $$\begin{aligned} \text {ES}_{q}\left( x\right) =\frac{1}{q}\intop _{0}^{q}\text {VaR}_{x}\,dx, \end{aligned}$$

for $0<q<1$ and VaR given by (32).

The *Tail Value at Risk* (TVaR), or tail conditional expectation (TCE), is the expected value of the loss in the event that it exceeds the VaR:

34 $$\begin{aligned} \text {TVaR}_{q}\left( x\right) =\frac{1}{1-q}\intop _{\text {VaR}_{q}}^{\infty }x\,f\left( x\right) dx \end{aligned}$$

By using Eq. (11), we get:

35 $$\begin{aligned} \text {TVaR}_{q}\left( x\right) =\frac{\alpha \left[ 1-q\right] ^{-1}}{(n+1)^{1/\beta +1}}\sum _{n=0}^{\infty }t_{n}\gamma \left( \frac{1}{\beta }+1,\left[ n+1\right] \left( \frac{\text {VaR}_{q}}{\alpha }\right) ^{\beta }\right) \end{aligned}$$

The *Tail Variance* (TV) is defined by the following expression:

36 $$\begin{aligned} \text {TV}_{q}\left( x\right) =E\left[ X^{2}|X>x_{q}\right] -\left[ \text {TVaR}_{q}\right] ^{2} \end{aligned}$$

Considering $$I=E\left[ X^{2}|X>x_{q}\right]$$, i.e.,

$$\begin{aligned} I=\text {TVaR}_{q}\left( x\right) =\frac{1}{1-q}\intop _{\text {VaR}_{q}}^{\infty }x^{2}\,f_{\text {BellW}}\left( x\right) dx, \end{aligned}$$

lead us to

37 $$\begin{aligned} I=\frac{\alpha ^{2}\left[ 1-q\right] ^{-1}}{(n+1)^{2/\beta +1}}\sum _{n=0}^{\infty }t_{n}\gamma \left( \frac{2}{\beta }+1,\left[ n+1\right] \left( \frac{\text {VaR}_{q}}{\alpha }\right) ^{\beta }\right) . \end{aligned}$$

By inserting Eqs. (35) and (37) in Eq. (36), we obtain the expression for TV for the CBellW model.

The *Tail Variance Premium* (TVP) combines information on both central tendency and dispersion. It is defined by

38 $$\begin{aligned} \text {TVP}_{q}\left( X\right) =\text {TVaR}_{q}+\delta \text {TV}_{q}, \end{aligned}$$

where $0<\delta <1$. By inserting Eqs. (36) and (35) in Eq. (38), we obtain the Tail Variance Premium for the CBellW model.

**Figure 12:**
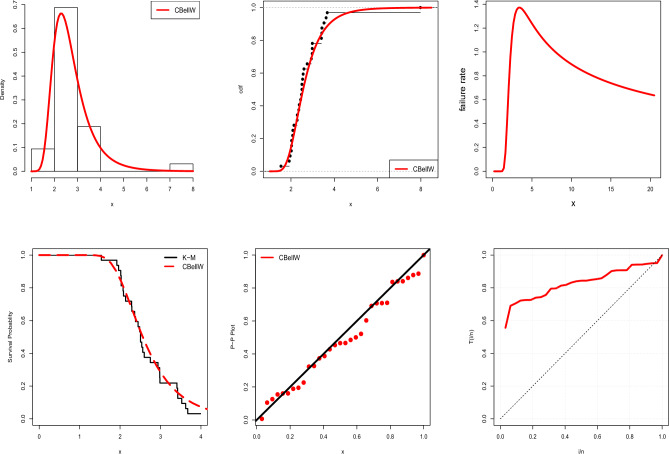
Estimated plots of pdf, cdf, hrf, K–M, P–P and TTT for Data-6.

In the following we exemplary apply VaR and ES to the UK auto-mobile collision claims data set. Various visualizations of the data set can be seen in Fig. 12. Table 10 gives the MLEs and SEs of the estimates for the fitted models. AIC, CAIC, BIC, and HQIC are shown in Table 11 along with other important metrics like *p*-values and the results of some tests (A, W, K–S).

**Table 10:** Fitted models with parameter estimates and standard errors.

Dist.	Est.	SE	Est.	SE	Est	SE	Est.	SE
CBellW $(\hat{\alpha }, \hat{\beta }, \hat{\lambda )}$	0.042	0.086	0.521	0.177	6.271	2.522	–	–
CPW $(\hat{\alpha }, \hat{\beta }, \hat{\lambda )}$	1.715	0.261	1.301	3.320	46.131	108.10	–	–
APW $(\hat{\alpha }, \hat{\beta }, \hat{\gamma )}$	0.005	0.002	3.401	0.290	0.002	0.002	–	–
TW $(\hat{\alpha }, \hat{\beta }, \hat{\lambda )}$	0.025	0.011	2.804	0.295	0.866	0.136	–	–
W$(\hat{\alpha }, \hat{\beta })$	0.062	0.025	2.460	0.270	–	–	–	–
EE$(\hat{\alpha }, \hat{\beta })$	1.695	0.253	56.40	33.149	–	–	–	–
BW$(\hat{\alpha }, \hat{\beta },\hat{a},\hat{b})$	0.263	0.004	2.366	0.004	2.495	0.771	0.368	0.075
W-Claim$(\hat{\alpha }, \hat{\beta }, \hat{\lambda )}$	2.506	0.411	0.896	0.122	0.003	0.001	–	–
MOW$(\hat{\alpha }, \hat{\beta },\hat{a})$	2.916	0.811	0.831	0.147	599.99	608.15	–	–
GW$(\hat{\alpha }, \hat{\beta }, \hat{\lambda )}$	12.741	1.421	0.505	0.003	21.198	2.381	–	–
GAPW$(\hat{\alpha }, \hat{\beta }, \hat{\gamma )}$	0.016	0.007	2.971	0.306	2.487	0.232	–	–

**Table 11:** Detailed summary of model selection measures.

Dist.	LL	AIC	CAIC	BIC	HQIC	A	W	K–S	*p*-value
CBellW	33.779	73.558	74.415	77.955	75.015	0.4519	0.0536	0.1033	0.8498
CPW	35.440	76.881	77.738	81.278	78.338	0.6549	0.0838	0.1265	0.6394
APW	40.756	87.512	88.369	91.909	88.970	1.4282	0.2074	0.1971	0.1452
TW	44.381	94.762	95.619	99.159	96.219	1.9915	0.2943	0.2190	0.0792
W	47.060	98.119	98.533	101.051	99.091	2.3974	0.3614	0.2340	0.0504
EE	35.358	74.716	75.130	77.648	76.068	0.6463	0.0823	0.1260	0.6440
BW	42.494	92.989	94.470	98.851	94.932	1.7868	0.2565	0.1999	0.1348
W-Claim	39.409	84.818	85.675	89.215	86.275	1.2052	0.1757	0.1658	0.3079
MOW	39.058	84.116	84.974	88.514	85.574	1.1407	0.1657	0.1682	0.2917
GW	41.215	88.429	89.286	92.827	89.887	1.1148	0.1527	0.2018	0.1281
GAPW	43.674	93.347	94.205	97.745	94.805	1.8795	0.2755	0.2107	0.1005

**Figure 13:**
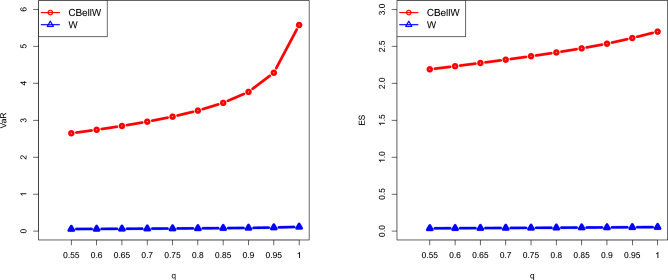
Graphical illustration of VaR and ES based on the MLEs.

Table 12 and Fig. 13 provide numerical and graphical representations, respectively, of both VaR and ES. By using the MLEs for the data set, the proposed CBellW model and the Weibull model are compared in terms of their VaR and ES. Note that a distribution is considered to have a heavier tail compared to another distribution when the associated risk measures yield larger values. Table 12 shows that the CBellW model has larger values of both risk measures than its counterpart, the Weibull model. Figure 13 also reveals that the proposed model has a heavier tail than the Weibull model. The readers are referred to Chan et al.^43^ for numerical computations of ES and VaR using the R package VaRES.

**Table 12:** VaR and ES for the CBellW and W model based on MLEs.

	*q*	0.55	0.60	0.65	0.70	0.75	0.80	0.85	0.90	0.95	0.99
VaR	CBellW	2.648	2.741	2.844	2.961	3.096	3.259	3.469	3.766	4.284	5.576
	W	0.057	0.060	0.063	0.067	0.071	0.075	0.080	0.087	0.097	0.115
ES	CBellW	2.189	2.231	2.274	2.319	2.366	2.416	2.472	2.535	2.612	2.698
	W	0.037	0.039	0.041	0.043	0.044	0.046	0.048	0.050	0.052	0.054

## Concluding remarks

In this paper, we have studied the CBellW model based on the CBell-G family of distributions. The failure rate function of the CBellW model can take different forms that makes it a very flexible and relevant model for real-world applications in numerous areas. We derived and discussed the key properties of the CBellW model in detail. The effectiveness of the CBellW model has been evaluated using real data applications (COVID-19, cancer, quality control, and actuarial data), and it has been compared with several established models. The conducted analysis revealed that the proposed CBellW model is superior to the competitors. The introduced distribution family represents a considerable contribution to the existing body of literature, given that it builds upon the DBellD as its foundation, inheriting the advantageous properties associated with Bell distributions. Hence, the proposed CBellW distribution family presents a promising alternative with the potential to outperform the well-established CP-G family. Since the quantile function of the CBellW model has a closed form solution it can be used to perform quantile regression analysis as an exemplary idea for fruitful future directions to further employ and enhance the CBellW model.

## Appendix

The cdf and pdf of the CBell-F family of distributions are given by

39 $$\begin{aligned} F(x)=\frac{\exp \left( e^{\lambda G(x)}-1\right) -1}{\exp \left( e^{\lambda }-1\right) -1} \end{aligned}$$

and

40 $$\begin{aligned} f(x)=\frac{\lambda \,g\left( x\right) \,\exp \left( \lambda \,G\left( x\right) \right) \,\exp \left( e^{\lambda G\left( x\right) }-1\right) }{\exp \left( e^{\lambda }-1\right) -1}, \end{aligned}$$

respectively, where *G*(*x*) and *g*(*x*) represent the baseline cdf and pdf.

### Useful expansion

A linear functional representation of the pdf and the cdf is useful to derive several structural and mathematical properties.

#### Proposition 2

A linear functional representation of the pdf and cdf is given by

41 $$\begin{aligned} f(x)=\sum _{v=0}^{\infty }\zeta _{v}l_{v+1}(x) \end{aligned}$$

and

42 $$\begin{aligned} F(x)=\sum _{v=0}^{\infty }\zeta _{v}L_{v+1}(x), \end{aligned}$$

respectively, where

43 $$\begin{aligned} \zeta _{v}=\frac{\lambda ^{v+1}}{v!\left( v+1\right) \left( \exp \left[ e^{\lambda }-1\right] -1\right) }\sum _{k=0}^{\infty }\sum _{r=0}^{\infty }\frac{\left( -1\right) ^{k+r}}{k!}\left( {\begin{array}{c}k\\ r\end{array}}\right) \left( 1+r\right) ^{v}, \end{aligned}$$

$L_{v+1}\left( x\right) =G(x)^{\left( v+1\right) }$ and $l_{v+1}\left( x\right) =\left( v+1\right) g\left( x\right) G\left( x\right) ^{\left( v+1\right) -1}$ are the cdf and pdf of the exp-G family with power parameters $(v+1)$, respectively.

#### Proof

To obtain the linear functional representation of the CBell-G family, we use the following two series, the binomial expansion and power series for the exponential functions, given as

44 $$\begin{aligned} (1-\omega )^{g}=\sum _{m=0}^{\infty }(-1)^{m}\,\left( {\begin{array}{c}g\\ m\end{array}}\right) \,\omega ^{m}, \end{aligned}$$

where $$\left( {\begin{array}{c}g\\ m\end{array}}\right)$$ represents the generalized binomial coefficients (the formula remains valid for $\omega$ such that $\left| \omega \right| <1$ only) and

45 $$\begin{aligned} \exp \left( -cx^{g}\right) =\sum _{m=0}^{\infty }\left( -1\right) ^{m}\,c^{m}\,\frac{x^{mg}}{m!} \end{aligned}$$

for any real numbers *c*, *g* and *x*. By using Eq. (45) to the last term of Eq. (40), one gets

46 $$\begin{aligned} \exp \left( e^{\lambda G\left( x\right) }-1\right) =\sum _{k=0}^{\infty }\frac{\left( -1\right) ^{k}}{k!}\left[ 1-e^{\lambda G\left( x\right) }\right] ^{k}, \end{aligned}$$

and applying Eq. (44) to Eq. (46) leads to

47 $$\begin{aligned} f(x)=\frac{\lambda \,g\left( x\right) \,}{\exp \left( e^{\lambda }-1\right) -1}\sum _{k=0}^{\infty }\sum _{r=0}^{\infty }\frac{\left( -1\right) ^{k+r}}{k!}\left( {\begin{array}{c}k\\ r\end{array}}\right) e^{\lambda G\left( x\right) \left[ 1+r\right] }. \end{aligned}$$

Expanding the last term of Eq. (47), we have

$$\begin{aligned} f(x)= & {} \frac{\lambda \,}{\left( \exp \left[ e^{\lambda }-1\right] -1\right) }\sum _{v=0}^{\infty }\sum _{k=0}^{\infty }\sum _{r=0}^{\infty }\frac{\left( -1\right) ^{k+r}}{k!v!\left( v+1\right) }\left( {\begin{array}{c}k\\ r\end{array}}\right) \left[ \lambda \left( 1+r\right) \right] ^{v}\times \\{} & {} \left( v+1\right) g\left( x\right) G^{\left( v+1\right) -1}\left( x\right) \end{aligned}$$

The desired mixture representation of *f*(*x*) is obtained. The expression for *F*(*x*) is obtained by integrating *f*(*x*). This completes the proof of Proposition 2. $\square$
