# Publisher Correction: The development of an extended Weibull model with applications to medicine, industry and actuarial sciences

**DOI:** 10.1038/s41598-024-64993-7

**Published:** 2024-06-20

**Authors:** Muhammad Imran, Najwan Alsadat, M. H. Tahir, Farrukh Jamal, Mohammed Elgarhy, Hijaz Ahmad, Arne Johannssen

**Affiliations:** 1 Department of Statistics, The Islamia University of Bahawalpur, Bahawalpur, 63100 Pakistan; 2 Department of Quantitative Analysis, College of Business Administration, King Saud University, P.O. Box 71115, 11587 Riyadh, Saudi Arabia; 3 Department of Basic Sciences, Higher Institute for Administrative Sciences, Belbeis, Al-Sharqia Egypt; 4 Mathematics and Computer Science Department, Faculty of Science, Beni-Suef University, Beni-Suef, 62521 Egypt; 5 Near East University, Operational Research Center in Healthcare, TRNC Mersin 10, Nicosia, 99138 Turkey; 6 Center for Applied Mathematics and Bioinformatics, Gulf University for Science and Technology, Mishref, Kuwait; 7 Department of Computer Science and Mathematics, Lebanese American University, Beirut, Lebanon; 8 Faculty of Business Administration, University of Hamburg, 20146 Hamburg, Germany

Correction to: *Scientific Reports* 10.1038/s41598-024-61308-8, published online 29 May 2024

The original version of this Article contained errors. In the Acknowledgements,

“This research was supported within the project RSPD2023R548, King Saud University, Riyadh, Saudi Arabia. The authors thank the reviewers for their valuable feedback that lead to a considerable improvement of this paper.”

now reads:

“This research is supported by researchers Supporting Project number (RSPD2024R548), King Saud University, Riyadh, Saudi Arabia. The authors thank the reviewers for their valuable feedback that lead to a considerable improvement of this paper.”

Additionally, in the Funding section,

“Open Access funding enabled and organized by Projekt DEAL.”

now reads:

“This research was supported within the project RSPD2024R548, King Saud University, Riyadh, Saudi Arabia. We acknowledge financial support from the Open Access Publication Fund of the University of Hamburg.”

Finally, in the Author Contributions section,

“Conceptualization: M.I. Data curation: N.A. Formal analysis: M.H.T. Validation: F.J., A.J. Writing—original draft: M.I., N.A. Writing—review editing: M.E., H.A. All the authors are agreed to publish this research work.”

now reads:

“Conceptualization: M.I., N.A. Methodology: N.A. Software: N.A. Validation: M.I., N.A., F.J. Formal analysis: M.H.T. Data curation: N.A. Investigation: M.I., N.A. Writing—original draft: M.I., N.A. Writing—review editing: M.E., H.A., A.J. Visualization: M.I., N.A. Supervision: M.E., A.J. Project administration: M.E., A.J.”

The original Article has been corrected.

